# Truncated Weibull–exponential distribution: methods and applications

**DOI:** 10.1038/s41598-023-48288-x

**Published:** 2023-11-27

**Authors:** Salman Abbas, Muhammad Farooq, Jumanah Ahmed Darwish, Saman Hanif Shahbaz, Muhammad Qaiser Shahbaz

**Affiliations:** 1 Department of Statistics, COMSATS University Islamabad, Lahore Campus, Lahore, Pakistan; 2 Department of Mathematics and Statistics, College of Science, University of Jeddah, Jeddah, Saudi Arabia; 3 Department of Statistics, King Abdulaziz University, Jeddah, Saudi Arabia

**Keywords:** Applied mathematics, Statistics

## Abstract

This paper introduces a truncated Weibull-exponential distribution and provides a thorough insight into its mathematical characteristics. These characteristics include moments, generating functions, inverse distribution function, and entropy. Various measures are also discussed about the distribution's reliability. A simulation study is carried out to assess the stability and consistency of the maximum likelihood estimates of the parameters. Finally, two social sciences data sets are used to assess the distribution's relevance in modeling real-world situations.

## Introduction

As the need to model real-world data problems continuously grows, researchers are expanding classical distributions to meet these new demands. To this end, several authors have proposed extensions to the existing families or classes of distributions, including the generalization of the parent distributions and introducing new models with some additional parameters. This ongoing development of the distribution theory reflects a continuous effort to improve the accuracy and applicability of the statistical models in a wide range of fields. Gupta et al.^1^ introduced a new "Exponentiated G-Class of distributions", which involves exponentiating the cumulative distribution function (*cdf*) to some positive power. Since then, many researchers have proposed different classes of distributions, including Marshall Olkin G–family by Marshall and Olkin^2^, the beta generalized-G family by Eugene et al.^3^, the Kumaraswamy–G family by Cordeiro and Nadarajah^4^, and the exponentiated generalized–G family by Cordeiro et al.^5^, a general method of generating continuous distributions by Alzaatrh et al.^6^, the Kumaraswamy Marshall Olkin–G family by Alizadeh et al.^7^, the transmuted exp–generalized–G family by Yousuf et al.^8^, and Kum–Transmuted–G family by Afify et al.^9^. These families of distributions provide several flexible and versatile models that can capture a wide range of data patterns and behaviors. Recently, Alzaatreh et al.^10^ have proposed a method to generate the families of distributions by using the truncated distribution and named the families of distributions as the truncated families of distributions. These families of distributions contain four different ways to generate the truncated families of distributions.

The Weibull distribution, a power transformation of the exponential distribution, was first studied by Fisher and Tippett^11^ in the context of the limiting distribution of extreme values in a sample. Since then, the Weibull distribution has been used in various applications, such as modeling the variability in diameter of powder particles by De Moivre^12^ and the failure rate properties of a system over time by Yong^13^. The Weibull distribution has been used to generate several new model for modeling of more complex phenomena. For instance, Alizadeh et al.^14^ have introduced the Transmuted Weibull–G family of distributions, Tahir et al.^15^ have derived a Weibull–G family of distributions, Cordeiro et al.^16^ have derived the exponentiated Weibull–G distributions, and Oluyede^17^ has proposed the Gamma Weibull–G family of distributions. These developments have expanded the applicability of the Weibull distribution and have provided more flexible models to capture a wider range of data patterns and behaviors.

Statistical models have been expanded to handle the complexity that arises in lifetime analysis. In certain situations, the domain of application of a phenomenon is reduced and one has to restrict the domain of the applicability of the underlying model. The truncated distributions play a useful role in such situations. The truncated distributions can be viewed as a form of a conditional distribution where the domain of the random variable is restricted under some conditions. When the domain of the random variable is restricted below a certain threshold, the resulting distribution is known as the left–truncated distribution. Conversely, if the domain is restricted above a specific threshold, the resulting distribution is a right-truncated distribution.

In this article, we have proposed a new truncated distribution called the truncated Weibull-exponential distribution, which is a truncated mixture of Weibull and exponential distribution. The proposed model is flexible and can be used for modeling data from various fields, including reliability sciences, social sciences, demography, and environmental sciences. We believe that the truncated Weibull-exponential distribution will provide a valuable addition to the existing models and will serve as an effective tool for data analysis in various fields.

The article structure follows. Section "Methodology" outlines the methodology used in this study. Section "The truncated Weibull–exponential distribution" presents the development of the truncated Weibull-Exponential model. Section "Properties of the distribution" contains important properties of the proposed distribution. Section "Reliability analysis" discusses some reliability measures. Section "Estimation" contains estimation of the model parameters. Section "Simulation study" is based on the simulation study to see the consistency of the estimation method. Some real data applications are given in Section "Applications". Section "Conclusions" contains the conclusions and findings of the study.

## Methodology

Mahdavi and Silva^18^ have derived a truncated family of distributions that can be used with any baseline distribution. The cumulative distribution function (*cdf*) and probability density function (*pdf*) of this new family are

1 $$G_{T} \left( x \right) = \frac{{H_{T} \left[ {F\left( x \right)} \right] - H_{T} \left( 0 \right)}}{{H_{T} \left( 1 \right) - H_{T} \left( 0 \right)}},$$

and

2 $$g_{T} \left( x \right) = \frac{{h_{t} \left[ {F\left( x \right)} \right]f\left( x \right)}}{{H_{T} \left( 1 \right) - H_{T} \left( 0 \right)}},$$

where $H_{T} \left( t \right)$ is *cdf* of any generator distribution, $H_{T} \left( 0 \right)$ and $H_{T} \left( 1 \right)$ are values of $H_{T} \left( t \right)$ at 0 and 1, $F\left( x \right)$ is *cdf* of any baseline distribution and $f\left( x \right)$ is the *pdf* corresponding to $F\left( x \right)$. Several distributions can be proposed by using different combinations of $H_{T} \left( t \right)$ and $F\left( x \right)$. Najarzadegan et al.^19^ have used the family (1) to propose a two parameter truncated Weibull-G family of distributions. Bantan et al.^20^ have proposed a truncated Burr-G family of distributions by using a Burr generator in (1). Almarashi et al.^21^ have used the Muth distribution as a generator in (1) to propose a truncated Muth-G family of distributions. In this paper, we have proposed a modified truncated Weibull-F family of distributions by using the following Weibull *cdf* and *pdf* as $H_{T} \left( t \right)$ and $h_{T} \left( t \right)$ in (1) and (2), respectively,

3 $$H_{T} \left( t \right) = 1 - \exp \left[ { - \left( {{t \mathord{\left/ {\vphantom {t \gamma }} \right. \kern-0pt} \gamma }} \right)^{k} } \right]\,\,;\,\,t,k,\gamma > 0$$

and

4 $$h_{T} \left( t \right) = \left( {{k \mathord{\left/ {\vphantom {k \gamma }} \right. \kern-0pt} \gamma }} \right)\left( {{t \mathord{\left/ {\vphantom {t \gamma }} \right. \kern-0pt} \gamma }} \right)^{k - 1} \exp \left[ { - \left( {{t \mathord{\left/ {\vphantom {t \gamma }} \right. \kern-0pt} \gamma }} \right)^{k} } \right]\,\,;\,\,t,k,\gamma > 0,$$

where $\gamma$ is the scale parameter and *k* is the shape parameter. Now, using (3) in (1), the *cdf* of the proposed truncated Weibull–F family of distributions is

5 $$G_{X} \left( x \right) = \frac{{1 - \exp \left[ { - \left\{ {{{F\left( x \right)} \mathord{\left/ {\vphantom {{F\left( x \right)} \gamma }} \right. \kern-0pt} \gamma }} \right\}^{k} } \right]}}{{1 - \exp \left( { - \gamma^{ - k} } \right)}}\,\,;\,\,x \in \Re \,,\,\gamma > 0\,,\,k > 0.$$

The density function corresponding to (5) is

6 $$g_{X} \left( x \right) = \left( {{k \mathord{\left/ {\vphantom {k \gamma }} \right. \kern-0pt} \gamma }} \right)f\left( x \right)\left[ {{{F\left( x \right)} \mathord{\left/ {\vphantom {{F\left( x \right)} \gamma }} \right. \kern-0pt} \gamma }} \right]^{k - 1} \exp \left[ { - \left\{ {{{F\left( x \right)} \mathord{\left/ {\vphantom {{F\left( x \right)} \gamma }} \right. \kern-0pt} \gamma }} \right\}^{k} } \right]\,\,;\,\,x \in \Re .$$

Various distributions can be derived by using different baseline distributions in (5) and (6). In the following, we have derived the truncated Weibull–Exponential distribution by using the exponential baseline distribution in (5) and (6).

## The truncated Weibull–exponential distribution

In this section, we have proposed the truncated Weibull–Exponential (*TWEx*) distribution by using the following *pdf* and *cdf* of the exponential distribution in (5) and (6).

$$F_{X} \left( x \right) = 1 - \exp \left( {{{ - x} \mathord{\left/ {\vphantom {{ - x} \lambda }} \right. \kern-0pt} \lambda }} \right)\,\,{\text{and}}\,\,f_{X} \left( x \right) = \left( {{1 \mathord{\left/ {\vphantom {1 \lambda }} \right. \kern-0pt} \lambda }} \right)\exp \left( {{{ - x} \mathord{\left/ {\vphantom {{ - x} \lambda }} \right. \kern-0pt} \lambda }} \right)\,\,;\,\,x,\lambda > 0$$.

The *cdf* of the proposed *TWEx* distribution is

7 $$G_{X} \left( x \right) = \left( {1 - {\text{e}}^{{ - \gamma^{ - k} }} } \right)^{ - 1} \left[ {1 - \exp \left\{ {{{ - \left( {1 - {\text{e}}^{{{{ - x} \mathord{\left/ {\vphantom {{ - x} \lambda }} \right. \kern-0pt} \lambda }}} } \right)} \mathord{\left/ {\vphantom {{ - \left( {1 - {\text{e}}^{{{{ - x} \mathord{\left/ {\vphantom {{ - x} \lambda }} \right. \kern-0pt} \lambda }}} } \right)} \gamma }} \right. \kern-0pt} \gamma }} \right\}^{k} } \right]\,\,;\,\,x,\gamma ,\lambda ,k > 0.$$

The density function corresponding to (8) is

8 $$g_{X} \left( x \right) = \frac{k}{{\lambda \gamma \left( {1 - {\text{e}}^{{ - \gamma^{ - k} }} } \right)}}\left( {\frac{{1 - {\text{e}}^{{ - {x \mathord{\left/ {\vphantom {x \lambda }} \right. \kern-0pt} \lambda }}} }}{\gamma }} \right)^{k - 1} \exp \left[ { - \left( {\frac{{1 - {\text{e}}^{{ - {x \mathord{\left/ {\vphantom {x \lambda }} \right. \kern-0pt} \lambda }}} }}{\gamma }} \right)^{k} } \right]{\text{e}}^{{ - \left( {{x \mathord{\left/ {\vphantom {x \lambda }} \right. \kern-0pt} \lambda }} \right)}} \,;\,x,\lambda ,\gamma ,k > 0.$$

It is interesting to note that if random variable *X* has density (8) then the distribution of $Y = \sqrt X$ has Weibull-Rayleigh distribution, given by Khalifa et al.^22^. The *TWEx* distribution provides some specific distributions as a special case. For example, for *k* = 1, the distribution reduces to a truncated exponential–exponential (*TEEx*) distribution, and for *k* = 2, it reduces to the truncated Rayleigh–exponential (*TREx*) distribution. It is to be noted that the proposed model is applicable for positive phenomenon only.

The density function of *TWEx* distribution can be easily expressed as the weighted sum of the exponentiated-exponential distributions as

9 $$g_{X} \left( x \right) = \sum\nolimits_{i = 0}^{\infty } {\sum\nolimits_{j = 0}^{\infty } {B_{i,j} \left( {k,\gamma } \right)\left( {{1 \mathord{\left/ {\vphantom {1 \lambda }} \right. \kern-0pt} \lambda }} \right){\text{e}}^{{{{ - \left( {i + 1} \right)x} \mathord{\left/ {\vphantom {{ - \left( {i + 1} \right)x} \lambda }} \right. \kern-0pt} \lambda }}} } } ,$$

where $$B_{i,j} \left( {k,\gamma } \right) = \frac{{\left( { - 1} \right)^{i + 1} \Gamma \left[ {2k\left( {j + 1} \right)} \right]}}{{i!j!\left( {1 - {\text{e}}^{{ - \gamma^{ - k} }} } \right)\Gamma \left[ {k\left( {j + 1} \right) - 1} \right]\gamma^{kj + 1} }}$$.

**Figure 1:**
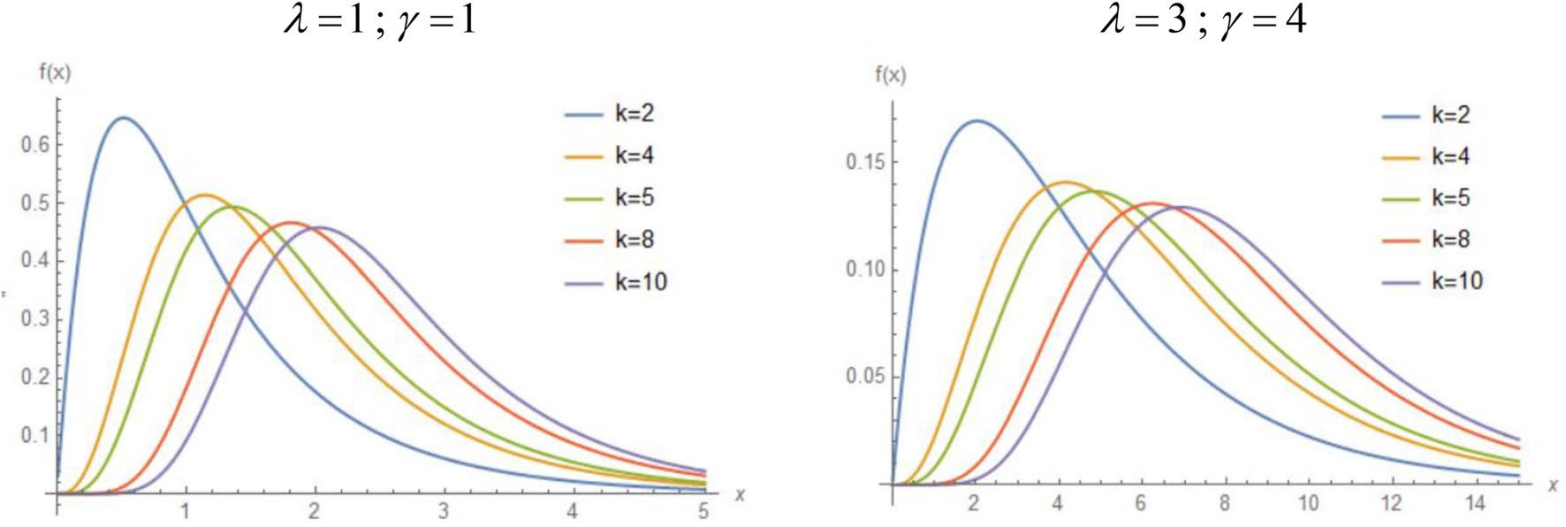
Plot of the density function of *TWEx* distribution for different values of the parameter.

The plots of the density function of *TWEx* distribution for specific values of *λ* and *γ* and for different choices of the parameter *k* are given in Fig. 1, below

From the above figure, we can see that the shape of the distribution changes with the value of *k*. We can see that the distribution approaches a symmetrical distribution for a large value of *k*. We can also see that the parameters $\lambda$ and $\gamma$ do not have a significant effect on the shape of the distribution. These parameters rather control the spread of the distribution.

### Shape of the distribution

The shape of the distribution is important in certain applications. In the following, we will obtain the mode of the distribution. For this, we first see that the logarithm of the density function is

10 $$\begin{aligned} \ln g_{X} \left( x \right) = & \ln k - \ln \lambda - \ln \gamma - \ln \left( {1 - {\text{e}}^{{ - \gamma^{ - k} }} } \right) - \left( {{x \mathord{\left/ {\vphantom {x \lambda }} \right. \kern-0pt} \lambda }} \right) + \left( {k - 1} \right)\ln \left[ {{{\left( {1 - {\text{e}}^{{{{ - x} \mathord{\left/ {\vphantom {{ - x} \lambda }} \right. \kern-0pt} \lambda }}} } \right)} \mathord{\left/ {\vphantom {{\left( {1 - {\text{e}}^{{{{ - x} \mathord{\left/ {\vphantom {{ - x} \lambda }} \right. \kern-0pt} \lambda }}} } \right)} \gamma }} \right. \kern-0pt} \gamma }} \right] \\ & \quad - \left[ {{{\left( {1 - {\text{e}}^{{{{ - x} \mathord{\left/ {\vphantom {{ - x} \lambda }} \right. \kern-0pt} \lambda }}} } \right)} \mathord{\left/ {\vphantom {{\left( {1 - {\text{e}}^{{{{ - x} \mathord{\left/ {\vphantom {{ - x} \lambda }} \right. \kern-0pt} \lambda }}} } \right)} \gamma }} \right. \kern-0pt} \gamma }} \right]^{k} . \\ \end{aligned}$$

The derivative of (10) with respect to *x* is

$$\frac{\partial }{\partial x}\ln g_{X} \left( x \right) = - \frac{1}{{\lambda \left( {1 - {\text{e}}^{{{{ - x} \mathord{\left/ {\vphantom {{ - x} \lambda }} \right. \kern-0pt} \lambda }}} } \right)}}\left[ {1 - k\left\{ {1 - \left( {\frac{{1 - {\text{e}}^{{{{ - x} \mathord{\left/ {\vphantom {{ - x} \lambda }} \right. \kern-0pt} \lambda }}} }}{\gamma }} \right)^{k} } \right\}} \right].$$

The mode is obtained as a solution of ${{\partial g_{X} \left( x \right)} \mathord{\left/ {\vphantom {{\partial g_{X} \left( x \right)} {\partial x}}} \right. \kern-0pt} {\partial x}} = 0$ and a unique mode of the distribution is readily obtained as

$$\hat{x} = - \lambda \ln \left[ {1 - \gamma \left( {1 - k^{ - 1} } \right)^{{{1 \mathord{\left/ {\vphantom {1 k}} \right. \kern-0pt} k}}} } \right].$$

The mode can be computed for specific values of the parameters.

## Properties of the distribution

In this section, some desirable properties of the proposed *TWEx* distribution are explored. These properties are studied in the following sub-sections.

### The quantile function

The quantile function is useful in obtaining percentiles of the distribution. The quantile function is obtained by solving $G\left( x \right) = m$ for *x*. Now, for the *TWEx* distribution, the quantile function is obtained by solving

$$\left( {1 - {\text{e}}^{{ - \gamma^{ - k} }} } \right)^{ - 1} \left[ {1 - \exp \left\{ { - \frac{{1 - {\text{e}}^{{{{ - x} \mathord{\left/ {\vphantom {{ - x} \lambda }} \right. \kern-0pt} \lambda }}} }}{\gamma }} \right\}^{k} } \right] = m$$

for *x* and is given as

11 $$Q_{X} \left( m \right) = - \lambda \ln \left[ {1 - \gamma \left\{ { - \ln \left[ {1 - \left( {1 - {\text{e}}^{{ - \gamma^{ - k} }} } \right)} \right]m} \right\}^{ - k} } \right].$$

The percentiles can be obtained by using any value of *m*, such that $m \in \left[ {0,1} \right]$, in (11). Specifically, the median can be obtained by using *m* = 0.5 in (11). Also, a random observation can be drawn from the *TWEx* distribution by replacing *m* with a uniform random number between [0,1] in (11).

### Moments and generating functions

The moments are useful in studying certain properties of a probability distribution. The *r*th moment of a random variable *X* having *cdf G*(*x*) is computed as

$$\mu_{r}^{/} = \int_{ - \infty }^{\infty } {x^{r} dG_{X} \left( x \right)} = \int_{ - \infty }^{\infty } {x^{r} g_{X} \left( x \right)dx} .$$

Now, using the density function of the *TWEx* distribution, from (9), in the above equation, we have

$$\mu_{r}^{/} = \int_{0}^{\infty } {\sum\nolimits_{i = 0}^{\infty } {\sum\nolimits_{j = 0}^{\infty } {B_{i,j} \left( {k,\gamma } \right)\left( {{1 \mathord{\left/ {\vphantom {1 \lambda }} \right. \kern-0pt} \lambda }} \right){\text{e}}^{{{{ - \left( {i + 1} \right)x} \mathord{\left/ {\vphantom {{ - \left( {i + 1} \right)x} \lambda }} \right. \kern-0pt} \lambda }}} } } dx} = \sum\nolimits_{i = 0}^{\infty } {\sum\nolimits_{j = 0}^{\infty } {B_{i,j} \left( {k,\gamma } \right)\int_{0}^{\infty } {\left( {{1 \mathord{\left/ {\vphantom {1 \lambda }} \right. \kern-0pt} \lambda }} \right){\text{e}}^{{{{ - \left( {i + 1} \right)x} \mathord{\left/ {\vphantom {{ - \left( {i + 1} \right)x} \lambda }} \right. \kern-0pt} \lambda }}} dx} } } .$$

Solving the integral, the *r*th moment of the *TWEx* distribution is

12 $$\mu_{r}^{/} = \sum\nolimits_{i = 0}^{\infty } {\sum\nolimits_{j = 0}^{\infty } {B_{i,j} \left( {k,\gamma } \right)\left( {i + 1} \right)^{{ - \left( {r + 1} \right)}} \lambda^{r} \Gamma \left( {i + 1} \right)} } .$$

The moments can be computed for specific values of *r* in (12).

The mean and variance of the distribution, for selected values of the parameters, are given in Table 1, below

**Table 1:** Mean and variance of *TWEx* distribution.

*k*	*λ*	Mean			Variance		
		$\gamma$			$\gamma$		
		3	4	5	3	4	5
2	2	1.963	2.275	2.599	0.722	1.001	1.455
	3	2.586	2.996	3.419	1.065	1.772	2.519
	4	3.402	3.943	4.504	1.508	2.443	3.452
3	2	2.083	2.493	2.905	0.875	1.500	2.240
	3	3.002	3.559	4.133	1.639	2.671	3.870
	4	4.250	5.011	5.822	2.506	4.050	5.799
4	2	2.236	2.702	3.189	1.041	1.819	2.671
	3	3.455	4.126	4.847	2.086	3.430	4.830
	4	5.087	6.083	7.142	3.299	5.461	7.776

From the table, we can observe that as the value of $\lambda$ increases, the mean of the *TWEx* distribution also increases. This is because $\lambda$ is the scale parameter of the distribution, and as it increases, the distribution shifts towards higher values, resulting in a larger mean. Similarly, we can see that as the parameter $\gamma$ increases, the mean of the *TWEx* distribution decreases. This is because $\gamma$ controls the rate at which the distribution decays, and as it increases, the distribution becomes more concentrated around lower values, resulting in a smaller mean. Finally, as *k* increases, the mean of the *TWEx* distribution also increases. This is because *k* is a shape parameter that controls the rate at which the tails of the distribution decay, and as it increases, the distribution becomes less heavy-tailed, resulting in a larger mean. The pattern for variance is almost the same as that of the mean.

The incomplete moment is another meaningful measure of a distribution. The incomplete moment of a distribution is defined as

$$\varphi_{p,y} = \int_{0}^{y} {x^{p} g_{X} \left( x \right)dx} .$$

Using (9), the incomplete moment for the *TWEx* distribution is given as

$$\varphi_{p,y} = \int_{0}^{y} {x^{p} \sum\nolimits_{i = 0}^{\infty } {\sum\nolimits_{j = 0}^{\infty } {B_{i,j} \left( {k,\gamma } \right)\left( {{1 \mathord{\left/ {\vphantom {1 \lambda }} \right. \kern-0pt} \lambda }} \right){\text{e}}^{{{{ - \left( {i + 1} \right)x} \mathord{\left/ {\vphantom {{ - \left( {i + 1} \right)x} \lambda }} \right. \kern-0pt} \lambda }}} } } dx} = \sum\nolimits_{i = 0}^{\infty } {\sum\nolimits_{j = 0}^{\infty } {B_{i,j} \left( {k,\gamma } \right)\left( {{1 \mathord{\left/ {\vphantom {1 \lambda }} \right. \kern-0pt} \lambda }} \right)\int_{0}^{y} {x^{p} {\text{e}}^{{{{ - \left( {i + 1} \right)x} \mathord{\left/ {\vphantom {{ - \left( {i + 1} \right)x} \lambda }} \right. \kern-0pt} \lambda }}} dx} } } .$$

Simplifying the integral, the *p*th incomplete moment for the *TWEx* distribution is

$$\varphi_{p,y} = \sum\nolimits_{i = 0}^{\infty } {\sum\nolimits_{j = 0}^{\infty } {B_{i,j} \left( {k,\gamma } \right)\left( {i + 1} \right)^{{ - \left( {p + 1} \right)}} \lambda^{p} \Gamma \left[ {\left( {p + 1} \right),{{\left( {i + 1} \right)y} \mathord{\left/ {\vphantom {{\left( {i + 1} \right)y} \lambda }} \right. \kern-0pt} \lambda }} \right]} } ,$$

where $\Gamma \left( {n,z} \right)$ is the incomplete gamma function defined as

$$\Gamma \left( {n,z} \right) = \int_{0}^{z} {w^{n - 1} {\text{e}}^{ - w} dw} .$$

The incomplete moments can be computed for specific values of *y* and *p*.

The moment generating function (*mgf*) is a useful function that can be used to compute moments of a distribution. The moment generating function of a continuous random variable *X* having density $f\left( x \right)$ is obtained as

$$M_{X} \left( t \right) = E\left( {{\text{e}}^{tX} } \right) = \int_{ - \infty }^{\infty } {xf\left( x \right)dx} .$$

Now, the *mgf* for the *TWEx* distribution is obtained as

$$\begin{gathered} M_{X} \left( t \right) = \int_{0}^{\infty } {{\text{e}}^{tx} \sum\nolimits_{i = 0}^{\infty } {\sum\nolimits_{j = 0}^{\infty } {B_{i,j} \left( {k,\gamma } \right)\left( {{1 \mathord{\left/ {\vphantom {1 \lambda }} \right. \kern-0pt} \lambda }} \right){\text{e}}^{{{{ - \left( {i + 1} \right)x} \mathord{\left/ {\vphantom {{ - \left( {i + 1} \right)x} \lambda }} \right. \kern-0pt} \lambda }}} } } dx} \hfill \\ \,\,\,\,\,\,\,\,\,\,\,\,\,\,\,\,\, = \sum\nolimits_{i = 0}^{\infty } {\sum\nolimits_{j = 0}^{\infty } {B_{i,j} \left( {k,\gamma } \right)\left( {{1 \mathord{\left/ {\vphantom {1 \lambda }} \right. \kern-0pt} \lambda }} \right)\int_{0}^{\infty } {{\text{exp}}\left[ {{{ - \left( {i + t\lambda + 1} \right)} \mathord{\left/ {\vphantom {{ - \left( {i + t\lambda + 1} \right)} \lambda }} \right. \kern-0pt} \lambda }} \right]dx} } } . \hfill \\ \end{gathered}$$

Solving the integral, the *mgf* of the *TWEx* distribution is

13 $$M_{X} \left( t \right) = \sum\nolimits_{i = 0}^{\infty } {\sum\nolimits_{j = 0}^{\infty } {B_{i,j} \left( {k,\gamma } \right)\left( {i - \lambda t + 1} \right)^{ - 1} } } .$$

The moments can be computed from (13).

### Entropy

The entropy is a useful measure of information. The Rényi^23^ entropy is computed as

$$H_{\kappa } \left( X \right) = \frac{1}{1 - \kappa }\ln \int_{0}^{\infty } {f^{\kappa } \left( x \right)dx} = \frac{\kappa }{1 - \kappa }\ln \int_{0}^{\infty } {f\left( x \right)dx} .$$

Using the density function of the *TWEx* distribution, the Rényi entropy is

$$H_{\kappa } \left( X \right) = \frac{\kappa }{1 - \kappa }\ln \int_{0}^{\infty } {\frac{k}{{\lambda \gamma \left( {1 - {\text{e}}^{{ - \gamma^{ - k} }} } \right)}}{\text{e}}^{{ - \left( {{x \mathord{\left/ {\vphantom {x \lambda }} \right. \kern-0pt} \lambda }} \right)}} \left( {\frac{{1 - {\text{e}}^{{ - \left( {{x \mathord{\left/ {\vphantom {x \lambda }} \right. \kern-0pt} \lambda }} \right)}} }}{\gamma }} \right)^{k - 1} \exp \left\{ { - \left( {\frac{{1 - {\text{e}}^{{ - \left( {{x \mathord{\left/ {\vphantom {x \lambda }} \right. \kern-0pt} \lambda }} \right)}} }}{\gamma }} \right)^{k} } \right\}dx} .$$

Transforming we have

$$H_{\kappa } \left( X \right) = \frac{\kappa }{1 - \kappa }\int_{0}^{1} {\ln \left[ {\frac{k}{{\gamma \left( {1 - {\text{e}}^{{ - \gamma^{ - k} }} } \right)}}\left( {\frac{1 - w}{\gamma }} \right)^{k - 1} \exp \left\{ { - \left( {\frac{1 - w}{\gamma }} \right)^{k} } \right\}} \right]dw}$$

Solving the integral, the entropy is

$$H_{\kappa } \left( X \right) = \frac{\kappa }{1 - \kappa }\left[ {\ln k - \ln \left\{ {\gamma \left( {1 - {\text{e}}^{{ - \gamma^{ - k} }} } \right)} \right\} - \left( {k - 1} \right)\left( {1 + \ln \gamma } \right) - \frac{{\gamma^{ - k} }}{1 + k}} \right].$$

The entropy can be computed for given values of the parameters.

## Reliability analysis

In this section, we have given some reliability analysis for the proposed *TWEx* distribution. The reliability analyses include the survival function, the hazard rate function, the moments of the residuals and the reversed residuals. These are given in the following sub-sections.

### Survival Function

The survival function is useful in reliability analysis. The function is used to obtain the probability that a component will be functioning after a specified time. The survival function is computed as $S\left( x \right) = 1 - F\left( x \right)$. The survival function for the *TWEx* distribution is given as

$$S_{X} \left( x \right) = \left( {1 - {\text{e}}^{{ - \gamma^{ - k} }} } \right)^{ - 1} \left[ {\exp \left\{ {{{ - \left( {1 - {\text{e}}^{{{{ - x} \mathord{\left/ {\vphantom {{ - x} \lambda }} \right. \kern-0pt} \lambda }}} } \right)} \mathord{\left/ {\vphantom {{ - \left( {1 - {\text{e}}^{{{{ - x} \mathord{\left/ {\vphantom {{ - x} \lambda }} \right. \kern-0pt} \lambda }}} } \right)} \gamma }} \right. \kern-0pt} \gamma }} \right\}^{k} - {\text{e}}^{{ - \gamma^{ - k} }} } \right]\,\,;\,\,x,\gamma ,\lambda ,k > 0.$$

The survival function can be computed for specific values of the parameters.

### Hazard rate function

The hazard rate function provides the information about instantaneous failure of a component that is still functioning at a specific time point. The hazard rate function for the *TWEx* distribution is obtained as

$$h\left( x \right) = \frac{g\left( x \right)}{{S\left( x \right)}} = \frac{{\left( {{k \mathord{\left/ {\vphantom {k {\lambda \gamma }}} \right. \kern-0pt} {\lambda \gamma }}} \right)\left\{ {{{\left( {1 - {\text{e}}^{{ - {x \mathord{\left/ {\vphantom {x \lambda }} \right. \kern-0pt} \lambda }}} } \right)} \mathord{\left/ {\vphantom {{\left( {1 - {\text{e}}^{{ - {x \mathord{\left/ {\vphantom {x \lambda }} \right. \kern-0pt} \lambda }}} } \right)} \gamma }} \right. \kern-0pt} \gamma }} \right\}^{k - 1} \exp \left\{ {{{ - \left( {1 - {\text{e}}^{{{{ - x} \mathord{\left/ {\vphantom {{ - x} \lambda }} \right. \kern-0pt} \lambda }}} } \right)} \mathord{\left/ {\vphantom {{ - \left( {1 - {\text{e}}^{{{{ - x} \mathord{\left/ {\vphantom {{ - x} \lambda }} \right. \kern-0pt} \lambda }}} } \right)} \gamma }} \right. \kern-0pt} \gamma }} \right\}^{k} {\text{e}}^{{ - {x \mathord{\left/ {\vphantom {x \lambda }} \right. \kern-0pt} \lambda }}} }}{{\left[ {\exp \left\{ {{{ - \left( {1 - {\text{e}}^{{{{ - x} \mathord{\left/ {\vphantom {{ - x} \lambda }} \right. \kern-0pt} \lambda }}} } \right)} \mathord{\left/ {\vphantom {{ - \left( {1 - {\text{e}}^{{{{ - x} \mathord{\left/ {\vphantom {{ - x} \lambda }} \right. \kern-0pt} \lambda }}} } \right)} \gamma }} \right. \kern-0pt} \gamma }} \right\}^{k} - {\text{e}}^{{ - \gamma^{ - k} }} } \right]}}.$$

**Figure 2:**
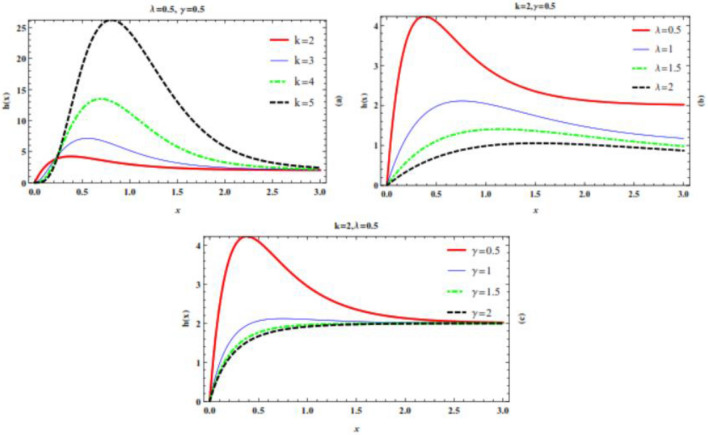
Plot of the hazard rate function for different values of the parameter.

The plots of the hazard rate function for different values of the parameters are given in Fig. 2 below

We can see that the hazard rate function of *TWEx* distribution has both increasing and decreasing trends.

### Moments of residual and reversed residual life

The residual life is useful to determine the life expectancy of components. The moments of residual and reversed residual life are useful in obtaining certain information about the life expectancy of the components. The *r*th moment of residual life is given as

$$\pi_{r} \left( t \right) = E\left[ {\left. {\left( {X - t} \right)^{r} } \right|X > t} \right] = \frac{1}{R\left( t \right)}\int_{t}^{\infty } {\left( {x - t} \right)^{r} g_{X} \left( x \right)dx} \,\,;\,\,r = 1,2,3, \ldots ,$$

where $R\left( t \right)$ is survival function associated with $g_{X} \left( x \right)$ at time *t*. Using the binomial expansion of $\left( {x - t} \right)^{r}$, we have

$$\pi_{r} \left( t \right) = E\left[ {\left. {\left( {X - t} \right)^{r} } \right|X > t} \right] = \frac{1}{R\left( t \right)}\sum\limits_{s = 0}^{r} {\left( { - 1} \right)^{r} \left( {\begin{array}{*{20}c} r \\ s \\ \end{array} } \right)t^{r} \int_{t}^{\infty } {x^{r - s} g_{X} \left( x \right)dx} } .$$

Now, using $g_{X} \left( x \right)$ from (9), the *r*th moment of residual life for the *TWEx* distribution is

$$\pi_{r} \left( t \right) = \frac{1}{R\left( t \right)}\sum\nolimits_{i = 0}^{\infty } {\sum\nolimits_{j = 0}^{\infty } {B_{i,j} \left( {k,\gamma } \right)\sum\limits_{s = 0}^{r} {\left( { - 1} \right)^{r} \left( {\begin{array}{*{20}c} r \\ s \\ \end{array} } \right)t^{r} \int_{t}^{\infty } {x^{r - s} \left( {{1 \mathord{\left/ {\vphantom {1 \lambda }} \right. \kern-0pt} \lambda }} \right){\text{e}}^{{{{ - \left( {i + 1} \right)x} \mathord{\left/ {\vphantom {{ - \left( {i + 1} \right)x} \lambda }} \right. \kern-0pt} \lambda }}} dx} } } } .$$

Solving the integral, the *r*th moment of residual life from the *TWEx* distribution is

$$\begin{aligned} \pi_{r} \left( t \right) = & \frac{1}{R\left( t \right)}\sum\nolimits_{i = 0}^{\infty } {\sum\nolimits_{j = 0}^{\infty } {B_{i,j} \left( {k,\gamma } \right)\sum\limits_{s = 0}^{r} {\left( { - 1} \right)^{r} \left( {\begin{array}{*{20}c} r \\ s \\ \end{array} } \right)t^{r} \left( {\frac{\lambda }{i + 1}} \right)^{r - s + 1} } } } \\ & \quad \times \left\{ {\Gamma \left( {r - s + 1} \right) - \Gamma \left[ {r - s + 1,{{\left( {i + 1} \right)t} \mathord{\left/ {\vphantom {{\left( {i + 1} \right)t} \lambda }} \right. \kern-0pt} \lambda }} \right]} \right\}. \\ \end{aligned}$$

The mean residual life can be obtained by using *r* = 1 in the above equation. The *r*th moment of reversed residual life is given as

$$\kappa_{r} \left( t \right) = E\left[ {\left. {\left( {X - t} \right)^{r} } \right|X < t} \right] = \frac{1}{F\left( t \right)}\int_{0}^{t} {\left( {x - t} \right)^{r} g_{X} \left( x \right)dx} \,\,;\,\,r = 1,2,3, \ldots .$$

Using the density function, given in (9), the *r*th moment of the reversed residual life for the *TWEx* distribution is given as

14 $$\kappa_{r} \left( t \right) = \frac{1}{F\left( t \right)}\sum\nolimits_{i = 0}^{\infty } {\sum\nolimits_{j = 0}^{\infty } {B_{i,j} \left( {k,\gamma } \right)\sum\limits_{s = 0}^{r} {\left( { - 1} \right)^{r} \left( {\begin{array}{*{20}c} r \\ s \\ \end{array} } \right)t^{r} \left( {\frac{\lambda }{i + 1}} \right)^{r - s + 1} \Gamma \left[ {r - s + 1,{{\left( {i + 1} \right)t} \mathord{\left/ {\vphantom {{\left( {i + 1} \right)t} \lambda }} \right. \kern-0pt} \lambda }} \right]} } } .$$

The mean residual life can be obtained by using *r* = 1 in (14).

## Estimation

In this section, we have discussed the maximum likelihood estimation of parameters of the *TWEx* distribution. For this, suppose that a sample of size *n* is available from the *TWEx* distribution. The log-likelihood function is then

15 $$\begin{aligned} \ell \left( {k,\gamma ,\lambda ;{\mathbf{x}}} \right) = & n\ln k - n\ln \gamma - n\ln \lambda - n\ln \left( {1 - e^{{ - \gamma^{ - k} }} } \right) - \frac{1}{\lambda }\sum\limits_{i = 1}^{n} {x_{i} } \\ & \quad + \left( {k - 1} \right)\sum\limits_{i = 1}^{n} {\ln \left[ {{{\left( {1 - {\text{e}}^{{{{ - x_{i} } \mathord{\left/ {\vphantom {{ - x_{i} } \lambda }} \right. \kern-0pt} \lambda }}} } \right)} \mathord{\left/ {\vphantom {{\left( {1 - {\text{e}}^{{{{ - x_{i} } \mathord{\left/ {\vphantom {{ - x_{i} } \lambda }} \right. \kern-0pt} \lambda }}} } \right)} \gamma }} \right. \kern-0pt} \gamma }} \right]} - \sum\limits_{i = 1}^{n} {\left[ {{{\left( {1 - {\text{e}}^{{{{ - x_{i} } \mathord{\left/ {\vphantom {{ - x_{i} } \lambda }} \right. \kern-0pt} \lambda }}} } \right)} \mathord{\left/ {\vphantom {{\left( {1 - {\text{e}}^{{{{ - x_{i} } \mathord{\left/ {\vphantom {{ - x_{i} } \lambda }} \right. \kern-0pt} \lambda }}} } \right)} \gamma }} \right. \kern-0pt} \gamma }} \right]^{k} } . \\ \end{aligned}$$

The derivatives of the log-likelihood function, (15), with respect to the unknown parameters are

16 $$\frac{\partial }{\partial k}\ell \left( {k,\gamma ,\lambda ;{\mathbf{x}}} \right) = n\left( {\frac{1}{k} - \frac{{\gamma^{ - k} \ln \gamma }}{{1 - {\text{e}}^{{ - \gamma^{ - k} }} }}} \right) + \sum\limits_{i = 1}^{n} {\ln \left( {\frac{{1 - {\text{e}}^{{{{ - x_{i} } \mathord{\left/ {\vphantom {{ - x_{i} } \lambda }} \right. \kern-0pt} \lambda }}} }}{\gamma }} \right)} - \sum\limits_{i = 1}^{n} {\left( {\frac{{1 - {\text{e}}^{{{{ - x_{i} } \mathord{\left/ {\vphantom {{ - x_{i} } \lambda }} \right. \kern-0pt} \lambda }}} }}{\gamma }} \right)^{k} \ln \left( {\frac{{1 - {\text{e}}^{{{{ - x_{i} } \mathord{\left/ {\vphantom {{ - x_{i} } \lambda }} \right. \kern-0pt} \lambda }}} }}{\gamma }} \right)}$$

17 $$\frac{\partial }{\partial \gamma }\ell \left( {k,\gamma ,\lambda ;{\mathbf{x}}} \right) = - \frac{nk}{\gamma } + \frac{{nk\gamma^{{ - \left( {k + 1} \right)}} {\text{e}}^{{ - \gamma^{ - k} }} }}{{1 - {\text{e}}^{{ - \gamma^{ - k} }} }} + \frac{k}{{\gamma^{k + 1} }}\sum\limits_{i = 1}^{n} {\left( {1 - {\text{e}}^{{{{ - x_{i} } \mathord{\left/ {\vphantom {{ - x_{i} } \lambda }} \right. \kern-0pt} \lambda }}} } \right)^{k} }$$

and

18 $$\frac{\partial }{\partial \lambda }\ell \left( {k,\gamma ,\lambda ;{\mathbf{x}}} \right) = - \frac{n}{\lambda } + \frac{1}{{\lambda^{2} }}\sum\limits_{i = 1}^{n} {x_{i} } - \frac{k - 1}{{\lambda^{2} }}\sum\limits_{i = 1}^{n} {\frac{{x_{i} {\text{e}}^{{{{ - x_{i} } \mathord{\left/ {\vphantom {{ - x_{i} } \lambda }} \right. \kern-0pt} \lambda }}} }}{{1 - {\text{e}}^{{{{ - x_{i} } \mathord{\left/ {\vphantom {{ - x_{i} } \lambda }} \right. \kern-0pt} \lambda }}} }}} + \frac{k}{{\lambda^{2} \gamma^{k} }}\sum\limits_{i = 1}^{n} {x_{i} {\text{e}}^{{{{ - x_{i} } \mathord{\left/ {\vphantom {{ - x_{i} } \lambda }} \right. \kern-0pt} \lambda }}} \left( {1 - {\text{e}}^{{{{ - x_{i} } \mathord{\left/ {\vphantom {{ - x_{i} } \lambda }} \right. \kern-0pt} \lambda }}} } \right)^{k - 1} }$$

The maximum likelihood estimators of *k*, *γ* and $\lambda$ can be obtained by equating the derivatives in (16–18) to zero and numerically solving the resulting equations.

The Fisher information matrix is useful to obtain the variances and covariances of the maximum likelihood estimators of the parameters. The entries of the Fisher information matrix for the *TWEx* distribution are given as

$${\mathbf{V}}^{ - 1} = - E\left[ {\begin{array}{*{20}c} {{{\partial^{2} \ell } \mathord{\left/ {\vphantom {{\partial^{2} \ell } {\partial k^{2} }}} \right. \kern-0pt} {\partial k^{2} }}} & {{{\partial^{2} \ell } \mathord{\left/ {\vphantom {{\partial^{2} \ell } {\partial k\partial \gamma }}} \right. \kern-0pt} {\partial k\partial \gamma }}} & {{{\partial^{2} \ell } \mathord{\left/ {\vphantom {{\partial^{2} \ell } {\partial k\partial \lambda }}} \right. \kern-0pt} {\partial k\partial \lambda }}} \\ {} & {{{\partial^{2} \ell } \mathord{\left/ {\vphantom {{\partial^{2} \ell } {\partial \gamma^{2} }}} \right. \kern-0pt} {\partial \gamma^{2} }}} & {{{\partial^{2} \ell } \mathord{\left/ {\vphantom {{\partial^{2} \ell } {\partial \gamma \partial \lambda }}} \right. \kern-0pt} {\partial \gamma \partial \lambda }}} \\ {} & {} & {{{\partial^{2} \ell } \mathord{\left/ {\vphantom {{\partial^{2} \ell } {\partial \lambda^{2} }}} \right. \kern-0pt} {\partial \lambda^{2} }}} \\ \end{array} } \right]$$

The entries of the Fisher information matrix are given in Appendix–A. The inverse of the Fisher information matrix provides the variance–covariance matrix of the maximum likelihood estimates, which help in obtaining the confidence intervals for the true population parameters.

## Simulation study

In this section, we have presented the simulation study to see the performance of the maximum likelihood estimates of the parameters. The simulation study has been conducted by generating random samples of different sizes from the *TWEx* distribution. The simulation algorithm is given below:

1. Generate random samples of sizes 50, 250, 500, 750, and 1000 from the *TWEx* distribution.
2. Compute maximum likelihood estimates of the parameters $\gamma ,\lambda$ and *k* for each sample.
3. Repeat steps 1 and 2 for 10,000 times.
4. Compute average estimates and the mean square error as

$$\overline{\hat{\theta }} = \frac{1}{10000}\sum\limits_{i = 1}^{10000} {\hat{\theta }_{i} } \,\,;\,\,MSE\left( {\hat{\theta }} \right) = \frac{1}{10000}\sum\limits_{i = 1}^{10000} {\left( {\hat{\theta }_{i} - \overline{\hat{\theta }}} \right)^{2} } \,\,;\,\,\hat{\theta } = \hat{\gamma }\,,\,\,\hat{\lambda }\,\,,\,\,\hat{k}$$ and $$\overline{\hat{\theta }} = \frac{1}{10000}\sum\limits_{i = 1}^{10000} {\hat{\theta }_{i} }$$.

The results of the simulation study are given in Table 2 below; with pre-specified values in the parenthesis;

**Table 2:** Estimated values of the parameters alongside the mean square errors.

Sample Sizes	Estimates			MSE		
	$k\left( {0.5} \right)$	$\lambda \left( {1.5} \right)$	$\gamma \left( {1.0} \right)$	$k\left( {0.5} \right)$	$\lambda \left( {1.5} \right)$	$\gamma \left( {1.0} \right)$
50	0.5180	1.5289	1.0384	0.0031	0.0064	0.0066
250	0.5055	1.5067	1.0098	0.0007	0.0004	0.0013
500	0.5029	1.5031	1.0044	0.0003	0.0002	0.0011
750	0.5022	1.5033	1.0050	0.0002	0.0002	0.0003
1000	0.5015	1.5020	1.0035	0.0001	0.0001	0.0003

	$k\left( {2.0} \right)$	$\lambda \left( {1.0} \right)$	$\gamma \left( {2.5} \right)$	$k\left( {2.0} \right)$	$\lambda \left( {1.0} \right)$	$\gamma \left( {2.5} \right)$
50	2.0182	1.0231	2.5336	0.0028	0.0019	0.0046
250	2.0043	1.0073	2.5084	0.0012	0.0009	0.0017
500	2.0028	1.0039	2.5063	0.0008	0.0006	0.0014
750	2.0017	1.0029	2.5036	0.0003	0.0004	0.0007
1000	2.0018	1.0023	2.5034	0.0002	0.0002	0.0003

	$k\left( {1.0} \right)$	$\lambda \left( {0.5} \right)$	$\gamma \left( {1.5} \right)$	$k\left( {1.0} \right)$	$\lambda \left( {0.5} \right)$	$\gamma \left( {1.5} \right)$
50	1.0162	0.5265	1.5436	0.0012	0.0029	0.0117
250	1.0042	0.5080	1.5097	0.0010	0.0011	0.0013
500	1.0027	0.5039	1.5050	0.0005	0.0007	0.0007
750	1.0018	0.5030	1.5039	0.0002	0.0005	0.0004
1000	1.0015	0.5023	1.5026	0.0002	0.0004	0.0002

From the above table, we can see that the estimated values of the parameters are close to the pre-specified values. Also, we can see that the mean square error of the estimates decreases with an increase in the sample size. The results of simulation study indicate that the maximum likelihood estimates of the parameters are consistent estimates.

## Applications

In this section, we have given two real data applications of the proposed *TWEx* distribution. The data set has been obtained from the Country Data Book^24^ and contains information about poverty level and college degrees. The summary measures of two data are given in Table 3, below

**Table 3:** Summary measures of the data.

Data	Max	Min	Mean	M.D	Variance	SD	Skewness	Kurtosis
Poverty	44.5	3.3	16.61	15.25	80.64	8.98	0.83	3.37
Degrees	30.7	5.6	11.99	11.30	13.90	3.73	2.04	10.72

We have fitted the following distributions; alongside the *TWEx*; distribution on these two data sets

- Weibull (W): $$g_{X} \left( x \right) = \frac{k}{\lambda }\left( {\frac{x}{\lambda }} \right)^{k - 1} \exp \left[ { - \left( {\frac{x}{\lambda }} \right)^{k} } \right]\,\,;\,\,x,k,\lambda > 0$$
- Exponential (E): $$g_{X} \left( x \right) = \frac{1}{\lambda }\exp \left( { - \frac{x}{\lambda }} \right)\,\,;\,\,x,\lambda > 0$$
- Log–Logistic (LL): $g_{X} \left( x \right) = \frac{{\gamma \lambda^{\gamma } x^{\gamma - 1} }}{{\left( {\lambda^{\gamma } + x^{\gamma } } \right)^{2} }}\,\,;\,\,x > 0\,\,,\,\,\lambda ,\gamma > 0$
- Exponentiated Weibull (EW): $g_{X} \left( x \right) = \frac{k\gamma }{\lambda }\left( {\frac{x}{\lambda }} \right)^{\gamma - 1} \exp \left[ { - \left( {\frac{x}{\lambda }} \right)^{\gamma } } \right]\left[ {1 - \exp \left\{ { - \left( {\frac{x}{\lambda }} \right)^{\gamma } } \right\}} \right]^{k - 1}$

The estimated model parameters alongside the values of  − 2(log-likelihood), Akaike Information Criterion (*AIC*), and the *p *value of Kolmogorov–Smirnov (*KS*) test are given in Table 4 below

**Table 4:** Fitted distributions on two data sets.

Data	Model	Parameter			$- 2\ell$	AIC	KS *p* value
		*k*	$\lambda$	$\gamma$			
I	TWEx	3.9947	8.0067	6.8446	563.200	569.200	0.948
	W	0.3021	1230.44		874.056	878.056	< 2.2 × 10^−16^
	E		16.6113		609.637	611.637	< 2.2 × 10^−16^
	LL		14.6855	2.9902	567.858	571.858	0.856
	EW	0.1112	908.073	2.1623	812.691	818.691	< 2.2 × 10^−16^
II	TWEx	19.4647	4.7121	0.9292	346.872	352.872	0.638
	W	0.3253	728.6940		704.008	708.008	< 2.2 × 10^−16^
	E		11.9904		473.814	475.814	< 2.2 × 10^−16^
	LL		11.5786	6.7044	357.411	361.411	0.432
	EW	0.1205	446.9910	2.2682	644.793	650.793	< 2.2 × 10^−16^

From the above table, we can see that the *TWEx* distribution is the best fit for the two data sets as it has the smallest value of *AIC* and the largest *p *value for the *KS*–test among all the competing distributions.

**Figure 3:**
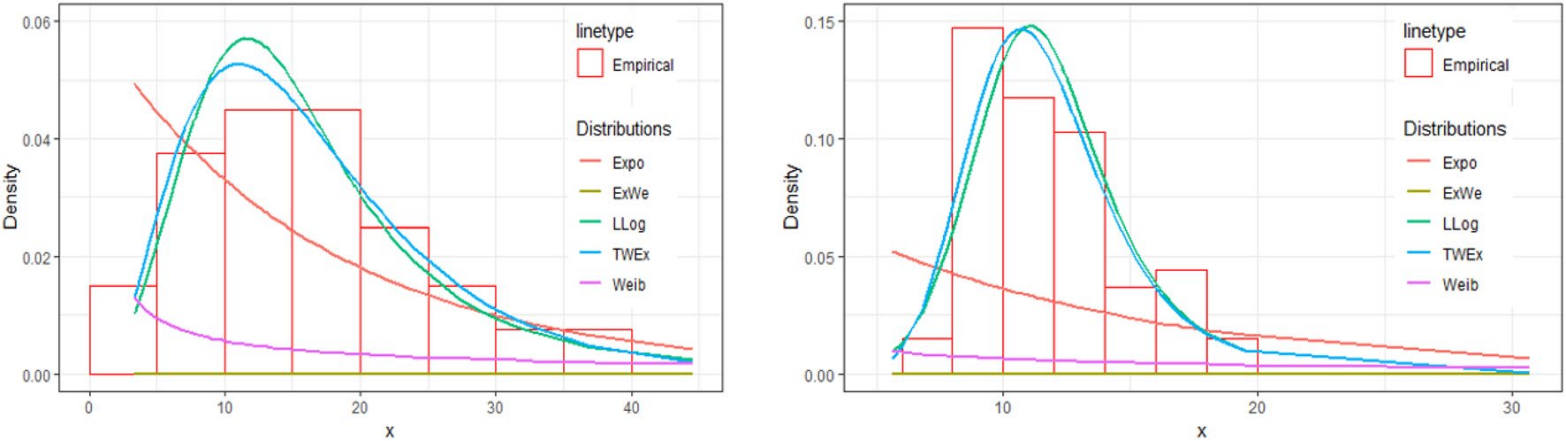
Density plots of various distributions for two data sets.

**Figure 4:**
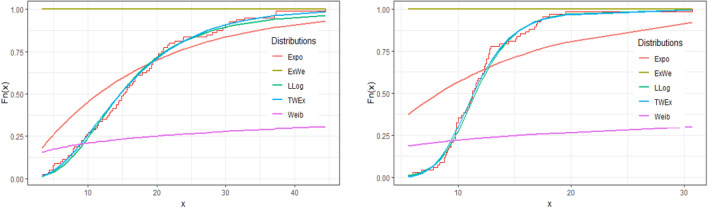
Distribution function plots of various distributions for two data sets.

**Figure 5:**
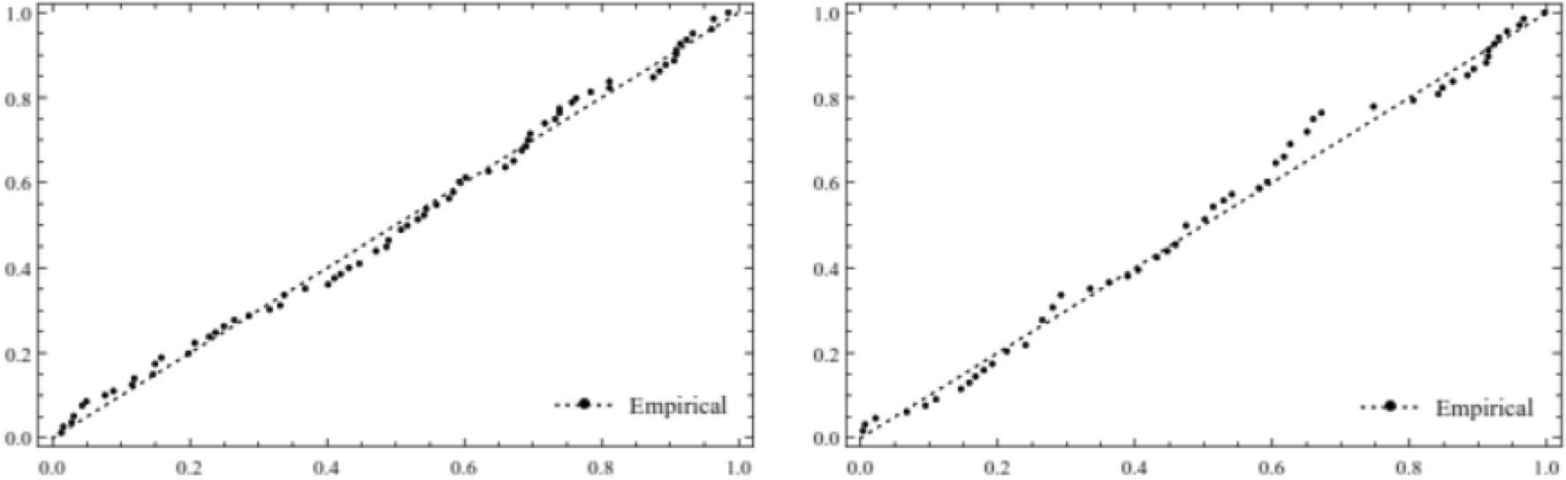
Probability plot of *TWEx* distribution for two data sets.

We have also constructed the plots of density and distribution functions of various distributions for the two data sets. These plots are given in Figs. 3 and 4 below. From these plots, we can see that the *TWEx* distribution is the best fit for the two data sets. Also, the log-logistic distribution is second best fit to the data. The other distributions used in the analyses are the poor fit. Further, the probability plot of the *TWEx* distribution for the two data sets, given in Fig. 5, shows the same result that the *TWEx* distribution is a reasonable fit for the two data sets.

## Conclusions

In this paper, we have proposed a *TWEx* distribution using the truncated Weibull distribution as a generator. Various desirable properties of the proposed *TWEx* distribution are explored. These properties include moments of the distribution, quantile function, entropy, and generating function. We have also discussed the reliability analysis for the proposed distribution. It has been observed that the proposed distribution has increasing and decreasing hazard rate functions. The moments of residual and reversed residual life has also been computed. We have also discussed the maximum likelihood estimation of the parameters for the proposed *TWEx* distribution. A simulation study is presented to see the performance of the estimates and it is found that the maximum likelihood estimates are consistent as the estimates converges to the true parameter values. Two real-life data applications of the proposed distribution have also been discussed and it is found that the proposed distribution is the best fit for the data in comparison with the other competing distributions.

## Data Availability

The data is available with the authors and can be obtained on request. Also, no medicine was tested on humans and animals for data analysis in this study.

## Appendix A

Entries of Fisher Information Matrix for *TWEx* distribution. We have assumed $1 - {\text{e}}^{{ - \gamma^{ - k} }} = z$ and $1 - {\text{e}}^{{{{ - x_{i} } \mathord{\left/ {\vphantom {{ - x_{i} } \lambda }} \right. \kern-0pt} \lambda }}} = w_{i}$

$$\begin{aligned} \frac{{\partial^{2} }}{{\partial k^{2} }}\ell \left( {k,\gamma ,\lambda ;{\mathbf{x}}} \right) = & - n\left[ {\frac{1}{{k^{2} }} + \frac{{\ln^{2} \gamma }}{{\gamma^{k} }}\left( {\frac{{{\text{e}}^{{ - \gamma^{ - k} }} }}{{\gamma^{k} z^{2} }} + \frac{1}{z}} \right)} \right] - \sum\limits_{i = 1}^{n} {\left( {\frac{{w_{i} }}{\gamma }} \right)^{k} \ln^{2} \left( {\frac{{w_{i} }}{\gamma }} \right)} \\ \frac{{\partial^{2} }}{\partial k\partial \gamma }\ell \left( {k,\gamma ,\lambda ;{\mathbf{x}}} \right) = & - \frac{n}{\gamma } + n\left[ {\frac{k}{{\gamma^{k} z}}\left( {\frac{{{\text{ln}}\gamma {\text{e}}^{{ - \gamma^{ - k} }} }}{{\gamma^{k + 1} z}} + \frac{{{\text{ln}}\gamma }}{\gamma z} - \frac{1}{kz}} \right)} \right] + \frac{1}{{\gamma^{2} }}\sum\limits_{i = 1}^{n} {\left[ {\left( {\frac{{w_{i} }}{\gamma }} \right)^{k} \left\{ {1 - \frac{k}{\gamma }\ln \left( {\frac{{w_{i} }}{\gamma }} \right)} \right\}} \right]} \\ \frac{{\partial^{2} }}{\partial k\partial \lambda }\ell \left( {k,\gamma ,\lambda ;{\mathbf{x}}} \right) = & - \frac{1}{{\lambda^{2} }}\sum\limits_{i = 1}^{n} {\frac{{x_{i} {\text{e}}^{{{{ - x_{i} } \mathord{\left/ {\vphantom {{ - x_{i} } \lambda }} \right. \kern-0pt} \lambda }}} }}{{w_{i} }}} + \frac{1}{{\lambda^{2} \gamma^{k} }}\sum\limits_{i = 1}^{n} {\left[ {x_{i} {\text{e}}^{{{{ - x_{i} } \mathord{\left/ {\vphantom {{ - x_{i} } \lambda }} \right. \kern-0pt} \lambda }}} w_{i}^{k - 1} \left\{ {1 + k\ln \left( {{{w_{i} } \mathord{\left/ {\vphantom {{w_{i} } \gamma }} \right. \kern-0pt} \gamma }} \right)} \right\}} \right]} \\ \frac{{\partial^{2} }}{{\partial \gamma^{2} }}\ell \left( {k,\gamma ,\lambda ;{\mathbf{x}}} \right) = & \frac{{nk^{2} {\text{e}}^{{ - \gamma^{k} }} }}{{\gamma^{{2\left( {k + 1} \right)}} }}\left( {\frac{{{\text{e}}^{{ - \gamma^{k} }} }}{{z^{2} }} + \frac{1}{z} + \frac{k + 1}{{kz}}} \right) + \frac{{k\left( {k + 1} \right)}}{{\gamma^{k} }}\sum\limits_{i = 1}^{n} {w_{i}^{k} } \\ \frac{{\partial^{2} }}{\partial \gamma \partial \lambda }\ell \left( {k,\gamma ,\lambda ;{\mathbf{x}}} \right) = & \frac{{k^{2} }}{{\lambda^{2} \gamma^{k + 1} }}\sum\limits_{i = 1}^{n} {x_{i} {\text{e}}^{{{{ - x_{i} } \mathord{\left/ {\vphantom {{ - x_{i} } \lambda }} \right. \kern-0pt} \lambda }}} w_{i}^{k - 1} } \\ \end{aligned}$$

and

$$\begin{aligned} \frac{{\partial^{2} }}{{\partial \lambda^{2} }}\ell \left( {k,\gamma ,\lambda ;{\mathbf{x}}} \right) = & \frac{{\left( {k - 1} \right)}}{{\lambda^{4} }}\sum\limits_{i = 1}^{n} {\left[ {\frac{{x_{i} {\text{e}}^{{{{ - x_{i} } \mathord{\left/ {\vphantom {{ - x_{i} } \lambda }} \right. \kern-0pt} \lambda }}} }}{{w_{i}^{2} }}\left( {2\lambda w_{i} - x_{i} w_{i} - x_{i} {\text{e}}^{{{{ - x_{i} } \mathord{\left/ {\vphantom {{ - x_{i} } \lambda }} \right. \kern-0pt} \lambda }}} } \right)} \right]} \\ & \quad - \frac{k}{{\gamma^{k} \lambda^{5} }}\sum\limits_{i = 1}^{n} {\left[ {x_{i} {\text{e}}^{{{{ - x_{i} } \mathord{\left/ {\vphantom {{ - x_{i} } \lambda }} \right. \kern-0pt} \lambda }}} w_{i}^{k - 2} \left\{ {2w_{i} + \left( {k - 1} \right)\lambda x_{i} {\text{e}}^{{{{ - x_{i} } \mathord{\left/ {\vphantom {{ - x_{i} } \lambda }} \right. \kern-0pt} \lambda }}} + \lambda x_{i} w_{i} } \right\}} \right]} \\ \end{aligned}$$
